# Preoperative Cardiac Diagnostics in Bariatric Patients with Diabetes and Perioperative Morbidity: Results of a Cohort of 258 Patients

**DOI:** 10.1007/s11695-021-05300-5

**Published:** 2021-03-03

**Authors:** Benjamin Stillhard, B. T. Truc Ngo, Ralph Peterli, Thomas Peters, Romano Schneider, Marko Kraljević, Marc Slawik, Bettina Wölnerhanssen

**Affiliations:** 1 Department of Internal Medicine, St. Claraspital Basel, CH-4002 Basel, Switzerland; 2 University of Basel, CH-4000 Basel, Switzerland; 3 St. Clara Research Ltd, St. Claraspital Basel, CH-4002 Basel, Switzerland; 4 Clarunis, Department of Visceral Surgery, University Centre for Gastrointestinal and Liver Diseases, St. Clara Hospital and University Hospital Basel, CH-4000 Basel, Switzerland

**Keywords:** Bariatric surgery, Preoperative examination, Diabetes, Coronary heart disease

## Abstract

**Purpose:**

The combination of obesity and diabetes mellitus are well-known risk factors for cardiovascular complications and perioperative morbidity in metabolic surgery. The aim of this study was to evaluate effectivity and reliability of the cardiac assessment in patients with diabetes prior to bariatric surgery.

**Setting:**

Private, university-affiliated teaching hospital, Switzerland

**Material and Methods:**

Retrospective analysis of prospectively collected data on results and consequences of cardiac assessments in 258 patients with obesity and diabetes scheduled for primary bariatric surgery at our institution between January 2010 and December 2018.

**Results:**

Out of 258 patients, 246 (95.3%) received cardiac diagnostics: 173 (67.1%) underwent stress-rest myocardial perfusion scintigraphy (MPS), 15 (5.8%) patients had other cardiac imaging including cardiac catheterization, 58 (22.5%) patients had echocardiography and/or stress electrocardiography, and 12 (4.7%) patients received no cardiac evaluation. Subsequently, cardiac catheterization was performed in 28 patients (10.9%), and coronary heart disease was detected and treated in 15 subjects (5.8%). Of these 15 individuals, 5 (33.3%) patients had diffuse vascular sclerosis, 8 (53.3%) patients underwent coronary angioplasty and stenting, and 2 (13.3%) patients coronary artery bypass surgery. Bariatric surgery was performed without perioperative cardiovascular events in all 258 patients.

**Conclusion:**

Our data suggest that a detailed cardiac assessment is mandatory in bariatric patients with diabetes to identify those with yet unknown cardiovascular disease before performing bariatric surgery. We recommend carrying out myocardial perfusion scintigraphy as a reliable diagnostic tool in this vulnerable population. If not viable, stress echocardiography should be performed as a minimum.

## Introduction

Numerous comorbidities are associated with obesity, including type 2 diabetes mellitus (T2DM), arterial hypertension, and dyslipidemia, together often referred to as metabolic syndrome [1, 2]. Furthermore, patients with obesity and diabetes are at significantly higher risk for coronary heart disease (CHD). Metabolic surgery has proven to be very effective in ameliorating or even remission of T2DM [3–6], leading to sustainable weight loss and reduction in comorbidities, including a significant reduction in cardiovascular events [7–9]. However, this population has an increased risk of perioperative morbidity, including cardiovascular events. Patient history may be misleading since patients with diabetes can suffer from silent CHD. So far, there is a lack of consensus concerning preoperative cardiac assessment in patients with severe obesity and diabetes. Established diagnostic pathways may differ between countries and institutions. A clear algorithm would help identify high-risk patients and improve their cardiovascular risk profile in the long term. The Obesity Surgery Mortality Risk Score (OS-MRS) predicts bariatric patients’ postoperative mortality of and can identify patients at higher risk [10]. However, diabetes is not included in OS-MRS, and the score fails to predict morbidity postoperatively [11]. The EOSS (Edmonton Obesity Staging System) helps identify patients with a higher risk of overall morbidity and describes bariatric patients’ complexity but is not a predictor for perioperative morbidity and mortality. The American Heart Association (AHA) suggests a cardiac and pulmonary algorithm assessment for elective non-cardiac surgery in patients with severe obesity [12]. In this algorithm, basic diagnostics include a 12-lead electrocardiography (ECG) and a chest X-ray for patients with at least one risk factor for CHD. The further cardiac evaluation depends on functional capacity. However, compared to basic diagnostic procedures (ECG, ECG stress test and (stress-) echocardiography), myocardial perfusion scintigraphy (MPS) improves the risk stratification for CHD in patients with obesity and diabetes considerably and is therefore particularly valuable [13–15]. MPS has a class I recommendation from the European heart association for the risk stratification in elective non-cardiac surgery [16]. In contrast, the effort and resources expended must be justified as well. This study aimed to evaluate effectivity, reliability, and consequences of the cardiac assessment in patients with diabetes before bariatric surgery at our center.

## Methods

This study was approved by the local ethical committee (Ethikkommission Nordwest- und Zentralschweiz, EKNZ, Switzerland). Informed consent was obtained from all patients. Indication for bariatric surgery in Switzerland changed during the study period. Before 2011, we operated on individuals with a BMI >40 kg/m or >35 kg/m^2^ with the presence of at least one comorbidity and failed conservative treatment over 2 years. Since 2011, indication for bariatric surgery includes a BMI >35 kg/m^2^ and failed conservative treatment of more than 2 years, or a BMI >50 kg/m^2^ and failed conservative treatment of more than 1 year. Individuals with an indication for bariatric surgery are assessed by a multidisciplinary team, including endocrinologists, dieticians, psychiatrists or psychologists, and surgeons. Additional specialists are involved individually depending on each patients’ specific needs, such as cardiologists, pneumologists, and gastroenterologists. Patients are committed to regular and lifelong follow-up care at a bariatric center.

In this trial, we retrospectively analyzed the results and consequences of cardiac assessments of 258 patients with class II/III obesity (WHO-Classification for obesity: class II defined as BMI of 35 to 39.9 kg/m^2^ and class III >40 kg/m^2^) and diabetes scheduled for primary bariatric surgery at our institution between January 2010 and December 2018. For this purpose, data up to 30 days follow-up was collected.

Minimal basic cardiac evaluation of all bariatric patients comprised of patient history and electrocardiogram. If an additional cardiovascular risk factor except for DM (e.g., arterial hypertension, positive family history, or dyslipidemia) was present, an ECG stress test was carried out. Smokers were advised to stop smoking or, if not possible, to reduce it to prevent perioperative complications. Routine screening for CHD in patients with obesity and diabetes was carried out by use of MPS. If the clinical presentation and the risk factors were strongly suggestive for CHD during the preoperative evaluation, patients were directly scheduled for invasive cardiac evaluation. If an adequate diagnostic or interventional cardiac procedure such as percutaneous coronary intervention (PCI), computed tomography of the coronary arteries, or stress magnetic resonance tomography of the heart had been performed within 1 year, we refrained from further cardiac testing. Patients of young age (less than 30 years) or a recent diagnosis of diabetes (less than 1 year) were not regularly scheduled for MPS. Limitations for MPS at our institution were a weight above 180 kg or patients’ inability to undergo MPS (e.g., claustrophobia or musculoskeletal disorders). If MPS was not performed, at least echocardiography and ECG stress test were carried out.

Single-photon emission computed tomography was performed with 99mTc-MIBI as a 2-day stress-rest imaging with either pharmacological stress (dipyridamole), or, if possible, ergometry. The device used for MPS was Symbia Intevo® by Siemens Healthcare GmbH, Germany, and for echocardiography Vivid E90® by General Electric Healthcare, GmbH, Germany. Interpretation was done by an experienced nuclear cardiologist and a nuclear radiologist. If the above-mentioned cardiovascular screening findings suggested a cardiopathy or CHD, cardiac catheterization, and, if necessary, angioplasty/stenting or coronary artery bypass surgery were performed. Bariatric surgery was realized under the inhibition of platelet aggregation (monotherapy). If a dual antiplatelet therapy was indicated, monotherapy was established 7 days pre-surgery, and a bridging with weight-adjusted low molecular heparin was established. The data used for this study was collected prospectively and analyzed retrospectively.

## Statistical Analysis

Categorical variables were summarized using counts and percentages. Continuous data were summarized using mean or median and standard deviation. The number needed to screen (NNS) is defined as the number of individuals required to be screened to detect one event. Statistical analysis was performed by IBM SPSS Statistics for Windows, Version 26.0. Armonk, NY: IBM Corporation.

## Results

Between January 2010 and December 2018, 1290 patients with class II/III obesity underwent primary bariatric surgery at our institution. Before surgery, 258 (20%) had a DM diagnosis and were included in this analysis. Three patients (1.2%) had DM type 1, and the remaining 255 patients had T2DM. In 200/258 patients (77.5%), the mean duration of diabetes was 7.9 (±7.4) years. In 58 patients, the duration of diabetes was not documented. The median Body Mass Index (BMI) at time of operation was 42 kg/m^2^ (±6.9), 80 (33%) patients were obesity class II and 178 (67%) class III, 48.1% were female, mean age was 52.7 years, and median HbA1c was 7.1% (±1.32). Cardiovascular comorbidities presented before screening were dyslipidemia in 219 (84.9%) patients, obstructive sleep apnea (OSA) in 99 (38.4%) patients, and arterial hypertension in 215 (83.3%) patients. Arterial hypertension was treated with medication in 207 patients (96.3% of 215), OSA was treated (e.g., continuous positive airway pressure) in 75 patients (75.8% of 99 patients), and dyslipidemia was treated in 137 patients (62.6% of 219). Complications of diabetes were neuropathy in 15 (5.8%), nephropathy in 11 (4.3%) patients, and known coronary heart disease in 17 (6.6%) patients. Retinopathy was not evident in any patient. Diabetes was not treated medically but using lifestyle-modifications (e.g., exercise and diet) in 33 patients (12.8%) (Table 1).

**Table 1:** Demographics

Total, *n*	258
Age, years (mean)	52.7
Female, *n* (%)	124 (48.1)
BMI, kg/m^2^, median (SD)	42.6 ±6.9
Roux-en-Y gastric bypass, *n* (%)	164 (63.6)
Sleeve gastrectomy, *n* (%)	94 (36.4)
Diabetes type 1, *n* (%)	3 (1.2)
Duration of DM (known in *n*=200), years, mean (Std.-deviation)	7.9 (±7.4)
**Comorbidities**	
Coronary heart disease, *n* (%)	17 (6.6)
1 vessel disease, *n*	4
> 1 vessel disease, *n*	11
Complication diabetes, *n* (%)	35 (13.6)
Neuropathy, *n*	15
Nephropathy, *n*	11
Retinopathy, *n*	0
>1, *n*	9
Dyslipidemia, *n* (%)	219 (84.9)
Obstructive sleep apnea, *n* (%)	99 (38.4)
Therapy (e.g., cpap), *n*	75
Arterial hypertension, *n* (%)	215 (83.3)
Stenosis carotis, *n* (%)	2 (0.8)
Stroke, *n* (%)	5 (1.9)
Peripheral artery disease, *n* (%)	1 (0.4)
Smoking status	
Never, *n* (%)	154 (59.7)
Ceased, *n* (%)	56 (21.7)
Active, *n* (%)	48 (18.6)
**Medication**	
Insulin only, *n* (%)	12 (4.7)
Antidiabetics, *n* (%)	141 (54.7)
Insulin and antidiabetics, *n* (%)	72 (27.9)
Therapy details*	
Short acting insulins, *n*	3
Long acting insulins, *n*	31
Short and long acting insulins, *n*	50
Glucagon-Like Peptide-1(GLP1) analogues, *n*	35
Sodium-Glucose Cotransporter 2 (SGTL-2) inhibitors, *n*	15
Biguanides, *n*	192
Sulfonylureas, *n*	39
Glitazones, *n*	23
Gliptins, *n*	48
No treatment of diabetes, *n* (%)	33 (12.8)
Acetylsalicylic acid, *n* (%)	61 (23.6)
Statin, *n* (%)	137 (53.1)
Antihypertensive medication, *n* (%)	207 (80.2)
Therapy details*	
Beta-blockers, *n*	82
Angiotensin-converting enzyme inhibitors, *n*	72
Angiotensin II receptor antagonists, *n*	111
Diuretics, *n*	126
Calcium channel blockers, *n*	75
Others, *n*	4
Anticoagulation oral, *n* (%)	12 (4.7)
**Blood levels**	
HbA1c (%), median (Std.-deviation)	7.1 ±1.3
Creatinine (mmol/l), median (Std.-deviation)	70 ±20
eGFR (CKD-EPI) (ml/min/1,73 m^2^), median ± SD	97 ±18.1
LDL (mmol/l), median ± SD	2.7 ±1.1
HDL (mmol/l), median ± SD	1.1 ±.05
Triglycerides (mmol/l), median ± SD	2.1 ±1.4

*BMI* Body Mass Index, *HbA1c* glycated hemoglobin, *cpap* continuous positive airway pressure, *eGFR* estimated glomerular filtration rate, *CKD-EPI* Chronic Kidney Disease Epidemiology Collaboration, *LDL* low-density lipoprotein, *HDL* high-density lipoprotein, *multiple naming possible, *SD* standard deviation. Values are expressed in mean, median, and standard deviation or numbers and percentage

A total of 246 of the 258 patients (95.3%) received preoperative cardiac diagnostics. One hundred seventy-three (67.1%) underwent stress-rest myocardial perfusion imaging. Nine (3.5%) patients did not receive an MPS due to technical limitations (weight >180 kg) or patients’ inability to perform MPS; instead, they underwent echocardiography and stress electrocardiography. A total of 58 (22.5%) patients received echocardiography and stress electrocardiography. Fifteen (5.8%) patients had had other recent cardiac imaging including diagnostic coronary angiography. Three patients (1.2%) were referred directly to cardiac catheterization due to clinical presentation. A total of nine (3.5%) patients had no specific cardiac workup before surgery as they were young (<30 years) and had a duration of type 2 diabetes of less than 1 year.

As a consequence of the before mentioned diagnostic procedures, cardiac catheterization was performed in a total of 28 patients (10.9% of 258 patients) before bariatric surgery. Of those patients, 24 (85.7% of 28 patients) received cardiac catheterization due to MPS findings. In one (3.6%) patient, pathological echocardiography and a treadmill-test were the indication, and in three (10.7%) patients, cardiac catheterization was performed due to clinical presentation during routine preoperative workup. Cardiac catheterization was scheduled timely, and delay to bariatric surgery was 4 (cardiac catheterization) to 6 weeks (coronary artery bypass surgery).

**Fig. 1:**
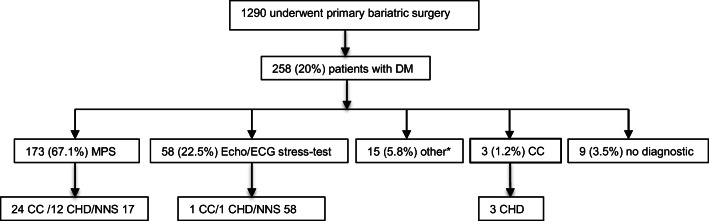
Diagnostic pathway. DM, diabetes mellitus; MPS, myocardial perfusion scintigraphy; ECG, electrocardiography; CC, cardiac catheterization; CHD, coronary heart disease; NNS, number needed to screen; *other = recent cardiac imaging, e.g., coronary computed tomogram. Numbers are expressed in count and percentage

A significant cardiovascular disease could be ruled out in 13 of the 28 patients who underwent cardiac catheterization. Five patients were newly diagnosed with diffuse vascular sclerosis but no relevant stenosis. Eight patients underwent coronary angioplasty and stenting, and two patients underwent coronary artery bypass surgery (Fig. 1). In patients with a diagnosed CHD, 2 (15.3%) were never-smokers, 5 (33.3%) were active smokers, and 8 (53.3%) smoked in the past.

The overall number needed to screen (= NNS) to detect one CHD was 17.2. Thereof, CHD was detected in 12 patients due to findings in the MPS (NNS 17). One patient with CHD was detected due to pathological TTE/Ergometry findings (NNS 58), and three patients with CHD were detected due to clinical presentation prior to cardiac catheterization. One patient with an already known CHD (one-vessel disease) underwent cardiac catheterization with angioplasty and stenting due to pathological findings in the MPS, resulting in the diagnosis of a two-vessel disease.

Laparoscopic Roux-en-Y gastric bypass was performed in 164 (63.6%) patients, and 94 (36.4%) received sleeve gastrectomy. In none of our patients, bariatric surgery had to be cancelled due to preoperative diagnostics, and bariatric surgery could be performed without any inhouse perioperative cardiovascular events. One patient (0.4%) died of an unknown cause at home 7 days after surgery. Relatives denied an autopsy. The preoperative diagnostics in this patient included an echocardiography and an ECG stress-test, both without pathological findings. Known comorbidities in this patient were arterial hypertension, OSA, and dyslipidemia. Another patient suffered from a transient ischemic attack (0.4%) perioperatively. Preoperative echocardiography and an ECG stress test in this patient had been without pathological findings. Known comorbidities were arterial hypertension, OSA, and dyslipidemia.

Estimated costs of the diagnostic procedures in Switzerland are as follows: The MPS with rest and pharmacological induced stress test costs around 2700 United States Dollars (USD). An ECG stress-test and transthoracic echocardiography are approximately 150 USD and 400 USD, respectively. In total, 173 MPS and 58 echocardiography plus ECG stress-test were performed to detect 15 cases of newly diagnosed CHD. The overall costs of our preoperative cardiac diagnostics in our cohort are therefore around 2000 USD per patient with class II/III obesity and diabetes.

## Discussion

Patients with obesity and diabetes scheduled for bariatric surgery are at high risk for perioperative complications [17]. This study aimed to evaluate the yield of screening for CHD before performing bariatric surgery in patients with class II/III obesity and diabetes. To our knowledge, there is no comparable publication in the preoperative cardiac workup for patients with obesity AND diabetes. CHD is known in approximately 5% of patients with obesity [18].

However, we newly diagnosed CHD in 14 (5.5%) patients and progression of an already known CHD in one patient due to preoperative cardiac workup. Therefore, in addition to individuals with previously known CHD (*n*=17), 12% (*n*=31) of our particular population had coronary heart disease as a severe comorbidity. This highlights the importance of a thorough preoperative workup. In none of our patients, bariatric surgery had to be cancelled, and perioperative performance during hospitalization was without cardiovascular events. The cause of one patient’s death within 30 days after surgery remains unclear. The etiology of the transient ischemic attack in another patient is nondistinctive as well. A cardiac involvement cannot be ruled out in both cases.

Coronary heart disease still is the cause of 19% of all deaths in Europe and responsible for an increase in hospitalization rate in most countries [19]. CHD is also the leading cause of mortality and morbidity in patients with DM [20]. In our retrospective analysis of 258 patients with obesity and diabetes, the NNS was 17 to detect one patient with CHD by MPS. In addition to a known low negative predictive value of MPS in asymptomatic patients with diabetes [21], this method seems to have significant relevance in the cardiac assessment of bariatric patients with diabetes. CHD is a known risk factor with an elevated odds ratio (1.4–1.5) for severe adverse events after bariatric surgery [18, 22]. We claim that the benefit of detecting and treating CHD before performing bariatric surgery in patients with diabetes outweighs the financial burden of the diagnostic procedures.

The International Federation for the Surgery of Obesity and Metabolic Disorders (IFSO) reports in its baseline demographic description that in Western Europe, 30% of the patients with obesity suffer from hypertension, 18% from dyslipidemia, and 20% from diabetes [23]. Our demographic data shows, however, that >80% of the population with obesity and diabetes also suffer from arterial hypertension and dyslipidemia, further increasing their cardiovascular risk profile. The metabolic syndrome leads to a higher perioperative risk in terms of cardiovascular events, but also anesthesia and intubation can be challenging [24–26]. However, patients with obesity with a higher risk for perioperative complications may have the highest benefit of bariatric surgery in the long term. Complications of obesity are manifold and are not only limited to the above-mentioned cardiovascular and metabolic burden. Also, the musculoskeletal system can be affected, there is a higher risk of developing various cancers, and individuals may suffer from socio-economic discrimination [27, 28]. Cardiac diagnostics in a population with obesity can be challenging. Overweight can be a technical limitation for the equipment, and it might impair the performance of echocardiography. In patients with obesity, it is often not possible (e.g., due to complications of the musculoskeletal system) to perform an adequate ECG stress test or stress echocardiography. Cardiac autonomic neuropathy resulting from DM can lead to silent ischemia and diagnosing CHD based on clinical observations only, leading to false negative results [29]. In many cases, more elaborate diagnostic imaging such as MPS, magnetic resonance imaging (MRI), computed tomography (CT), or nuclear medicine (NM) imaging with PET or SPECT is mandatory.

### Limitations

This investigation is a retrospective analysis without a control group, and there is a lack of generalizability due to its descriptive nature.

The discrepancy of the numbers of myocardial perfusions scans for screening for CHD, and the aim of our screening algorithm (every patient with obesity and diabetes undergoing MPS) is apparent. This is partly explained by using MPS continuously only from 2012 onwards, but patients were included in this study from 2010 to 2018. A higher detection rate of CHD could be the consequence. The follow-up presented in this study is 30 days, but a longer period could probably unmask a higher CHD prevalence. Differences in health care systems in various countries could impair the comparison of procedural costs.

## Conclusion

Our data suggest that a thorough cardiac assessment is mandatory in bariatric patients with diabetes to identify those with yet unknown cardiovascular disease before performing bariatric surgery. Our findings might help develop an algorithm for the preoperative assessment in patients with class II/III obesity and diabetes. We recommend carrying out myocardial perfusion scintigraphy as a reliable diagnostic tool in this vulnerable population. If not viable, stress echocardiography should be performed as a minimum. Further investigations, prospective and randomized, are required to establish recommendations for preoperative diagnostics in bariatric patients with diabetes.

## Declarations

The protocol was submitted and approved by the Local Research and Ethics Committee.
